# High-Dimensional Immune Profiling Reveals Innate-Adaptive Rebalancing and Subpopulation Trajectories in Pediatric Severe *Mycoplasma pneumoniae* Pneumonia

**DOI:** 10.3390/diagnostics16152429

**Published:** 2026-07-31

**Authors:** Chen Shen, Deze Li, Xiaotong Wang, Yang Sun, Huiwen Zheng, Hao Chen, Xin Ni, Shunying Zhao

**Affiliations:** National Center for Children’s Health, Beijing Children’s Hospital, Capital Medical University, Beijing 100045, China; shenchen1110@126.com (C.S.); li_deze@163.com (D.L.); wxt_medical@163.com (X.W.); 18098009285@163.com (Y.S.); hanhui820@126.com (H.Z.); chenhaosen0208@163.com (H.C.)

**Keywords:** *Mycoplasma pneumoniae* pneumonia, immune profiling, monocytes, T-cell exhaustion, innate-adaptive balance, biomarkers, pleural effusion

## Abstract

**Background**: Severe *Mycoplasma pneumoniae* pneumonia (SMPP) presents with heterogeneous clinical complications and imaging manifestations, including pleural effusion (PE), bronchiolitis obliterans (BO), and distinct imaging/bronchoscopic phenotypes. The precise systemic immune subpopulation dynamics underlying these distinct phenotypes remain poorly defined. **Methods**: We conducted flow cytometric profiling of peripheral blood mononuclear cells (PBMCs) from pediatric patients to compare mild MPP (MMPP-N) and severe MPP subtypes. SMPP patients were stratified into subgroups including those with BO (SMPP&BO, *n* = 3), PE (SMPP&PE), and three imaging/bronchoscopic phenotypes: Group A (SMPP-GA, mucous plugs), Group B (SMPP-GB, mucosal necrosis), and Group C (SMPP-GC, diffuse bronchiolitis). Composite immune indices and multi-marker discriminant analysis were constructed for secondary analysis. **Results**: Comprehensive high-dimensional profiling suggested that a fundamental innate-adaptive immune rebalancing may represent a key pathophysiological axis in severe disease. Patients with PE exhibited massive CD16^−^ monocyte expansion (Delta = 40.0%) coupled with CD8^+^ Teff collapse (Delta = −9.2%). Preliminary data also suggest BO may be marked by CD56^−^CD11C^+^ monocyte depletion (Delta = −23.1%, *n* = 3), warranting further validation. Imaging subgroup profiling revealed that SMPP-GC featured major B-cell maturation alterations (HLADR^+^CD45RA^high^ expansion and CD38^+^CD25^low^ depletion). Composite indices including the Adaptive Exhaustion Score suggested discriminatory power (AUC = 0.90 for PE vs. uncomplicated SMPP). The Innate-Adaptive Ratio increased progressively with severity (Spearman rho = +0.40, *p* = 0.0002). **Conclusions**: SMPP is not immunologically uniform. Innate-adaptive rebalancing underlies complication-specific phenotypes: inflammatory monocyte mobilization with adaptive exhaustion is strongly associated with PE, whereas B-cell maturation alterations characterize diffuse bronchiolitis. These findings identify potential biomarker candidates for patient stratification, though small-cohort observations require extensive validation in larger prospective studies.

## 1. Introduction

*Mycoplasma pneumoniae* (MP) is one of the most prevalent atypical pathogens causing community-acquired pneumonia (CAP) in children and adolescents worldwide, accounting for 10–40% of all CAP cases depending on geographic region, seasonality, and circulating strain prevalence [1,2]. Although the majority of MP infections present as mild, self-limiting upper respiratory tract illnesses, a clinically significant subset of patients progresses to severe pneumonia requiring hospitalization, intensive respiratory support, and occasionally life-saving interventions [3]. Over the past decade, the global emergence and spread of macrolide-resistant MP strains has further compounded the clinical challenge, leading to prolonged disease courses, treatment failures, and increased rates of severe complications [4]. The delayed re-emergence of MPP following COVID-19 pandemic restrictions, with widespread reports of increased severity and macrolide refractoriness during the 2023–2024 resurgence, has further underscored the urgent need for improved immunopathological understanding [5,6].

Severe *Mycoplasma pneumoniae* pneumonia (SMPP) is a heterogeneous clinical entity encompassing a broad spectrum of imaging manifestations. Pleural effusion (PE) represents one of the most common complications, occurring in up to 20% of hospitalized SMPP cases and significantly extending hospital stays [7]. Bronchiolitis obliterans (BO), although less frequent, carries substantial long-term morbidity due to irreversible airway obstruction and progressive pulmonary function decline [8]. Additionally, bronchoscopic evaluation and imaging findings of SMPP patients reveal diverse intraluminal pathologies, including massive mucous plug formation, mucosal erosion and necrosis, and diffuse bronchiolitis, suggesting that distinct pathophysiological processes underlie these phenotypically divergent presentations [9].

The pathogenesis of SMPP is increasingly understood as being driven by dysregulated host immune responses rather than direct bacterial cytotoxicity [10]. MP lacks a cell wall and adheres to respiratory epithelium through specialized attachment organelles, triggering robust innate and adaptive immune cascades [11]. T lymphocytes, particularly CD4^+^ and CD8^+^ subsets, are critical orchestrators of the anti-MP response, while innate immune compartments including monocytes, dendritic cells, NK cells, and B cells contribute to both protective immunity and immunopathology [12]. However, comprehensive characterization of how specific immune subpopulations restructure across the full severity and complication spectrum of SMPP remains conspicuously lacking. MP-HSP (Henoch-Schonlein purpura with Mycoplasma infection) was included as an immunologically distinct comparator within the MP infection spectrum, not as an SMPP phenotype. HSP represents a well-characterized MP-related immune-mediated vasculitis, providing an external anchor to differentiate SMPP-specific immune changes from generic MP-related immune dysregulation. Analyses with and without MP-HSP yielded directionally concordant primary findings.

Recent advances in multiparameter flow cytometry have enabled high-dimensional immune profiling at single-cell resolution, revealing disease-specific immune signatures with diagnostic, prognostic, and therapeutic implications in respiratory infections [13,14]. In the context of MP infection, prior studies have documented selective T-cell subset alterations and cytokine dysregulation in severe disease; however, these investigations have typically been limited in panel breadth, restricted to binary severe-versus-mild comparisons, and have not systematically correlated immune profiles with specific imaging phenotypes [15,16].

In this study, we employed an extensive multiparameter flow cytometry panel to systematically quantify 74 immune cell populations in peripheral blood from pediatric patients across ten clinically defined MPP groups. By integrating detailed imaging and bronchoscopic phenotyping with high-dimensional immune characterization, we aimed to provide a detailed immune profile of SMPP and explore the potential pathophysiological axes associated with distinct complications. We advance beyond traditional single-marker comparisons by constructing composite immune indices, performing severity-gradient correlation analysis, and conducting multi-marker discriminant analysis to investigate the shared immunological patterns underlying SMPP heterogeneity.

## 2. Materials and Methods

### 2.1. Patient Recruitment

Participants meeting the eligibility criteria for pneumonia satisfied one or more of the following: (1) patients diagnosed with MP infection or single-pathogen infection without MP infection, aged 3–10 years; (2) samples were collected during the acute phase of the disease course (7–10 days after onset). Participants meeting any of the following criteria were excluded: (1) patients with pneumonia complicated by multiple pathogens; (2) patients with severe malnutrition or immunodeficiency; (3) patients with severe primary diseases of the heart (congenital heart disease, myocarditis, etc.), liver (aspartate aminotransferase or alanine aminotransferase ≥ 1.5 times the upper limit of normal), kidney (blood urea nitrogen > 8.2 mmol/L, serum creatinine > 104 µmol/L), hematopoietic system (moderate to severe anemia), or mental illness; (4) patients currently using immunosuppressive agents or modulators such as glucocorticoids.

### 2.2. Patient Grouping

The differential diagnosis between MPP and non-MPP (NMPP) followed our previous study: (1) serum anti-MP IgM titer ≥ 1:320 (latex agglutination test); (2) positive MP RNA result from RT-PCR of a pharyngeal swab. MPP was confirmed when both criteria were satisfied. If neither was met, NMPP was diagnosed.

According to our previous study, MPP is classified into mild MPP (MMPP) and severe MPP (SMPP): (1) MMPP: high fever lasting less than 5 days, without wheezing or dyspnea; imaging findings showed patchy or cloud-like shadows or consolidation involving less than half of a lung lobe. Bronchoscopy revealed scant airway mucus secretion, without mucosal erosion or necrosis, and routine laboratory results were normal or mildly elevated. (2) SMPP: persistent high fever for more than 5 days, hypoxemia with wheezing or dyspnea, imaging findings of high-density consolidation involving multiple lobes or more than two-thirds of a single lobe, or diffuse bronchiolitis as the main radiographic feature; bronchoscopy showed bronchial mucosal erosion, necrosis, and often mucus plug formation. Routine laboratory indicators were markedly elevated. Given the marked heterogeneity in imaging findings, SMPP was further divided into three types: (1) SMPP-group A (SMPP-GA): predominant imaging findings of large consolidation, with airway mucus hypersecretion leading to mucus plugging or plastic bronchitis under bronchoscopy, no obvious mucosal necrosis, and no significant elevation in routine laboratory markers; (2) SMPP-group B (SMPP-GB): some children presented with markedly increased C-reactive protein (CRP), and bronchoscopy primarily revealed mucosal erosion and necrosis, often accompanied by mucus plugs; (3) SMPP-group C (SMPP-GC): imaging mainly showed diffuse bronchiolitis, often with hypoxemia, and bronchoscopy typically revealed scant airway mucus secretion without mucosal erosion or necrosis.

For certain cross-group comparisons, severe MPP patients were aggregated as SMPP-ALL, combining all severe complication subgroups (SMPP&BO, SMPP&PE, SMPP-N, SMPP-GA, SMPP-GB, SMPP-GC) to increase statistical power for severe-versus-moderate comparisons. This aggregated group definition is explicitly stated wherever SMPP-ALL is used in Section 3. A detailed flowchart of patient enrollment, severity classification, complication assignment, and imaging/bronchoscopic phenotyping is provided in Figure S6, with an accompanying group membership table (Table S1) clarifying mutually exclusive versus overlapping subgroup definitions. Analyses involving SMPP-ALL use the aggregated severe group (*n* = 42); analyses by complication use mutually exclusive subsets (SMPP-N, SMPP&PE, SMPP&BO); analyses by imaging/bronchoscopic grade use the 18 phenotyped patients (SMPP-GA, SMPP-GB, SMPP-GC) (Table 1; Figure S6).

### 2.3. Peripheral Blood Mononuclear Cells

Peripheral blood (4–6 mL) was collected from each child into K3-EDTA tubes. Density gradient centrifugation was performed, after which the cells were washed and resuspended in culture medium containing dimethyl sulfoxide (DMSO). The samples were then slowly frozen at −80 °C before transferring to liquid nitrogen for storage. Viability of the isolated peripheral blood mononuclear cells (PBMCs) was assessed by trypan blue staining after thawing. All analyzed samples showed post-thaw viability of ≥80%, as determined by trypan blue exclusion.

### 2.4. Multicolor Flow Cytometry

Up to 0.5 × 10^6^ cells were first incubated with 10% fetal bovine serum (FBS) in 1× PBS for 10 min. Subsequently, the cells were stained at room temperature for 20 min in 100 μL of PBS containing 0.1 μL of fixable viability dye eFluor 506 (Thermo Fisher Scientific, Waltham, MA, USA), 2 μL of FcR blocking reagent, and the antibody cocktail listed in Table S2. After two washes with PBS, the cells were resuspended in 100 μL of MACS buffer (PBS containing 2 mM EDTA, pH 8.0, and 0.5% bovine serum albumin [BSA]) and stored in the dark at 4 °C for up to 4 h until flow cytometry acquisition.

Following FSC/SSC gating and live single-cell sorting (Figure S1A), peripheral blood mononuclear cells (PBMCs) were classified into T-cells (CD3^+^CD20^−^), B cells (CD3^−^CD20^+^), and non-T/B cells (CD3^−^CD20^−^) (Figure S1B). The latter population included dendritic cells (DCs), natural killer (NK) cells, myeloid cells, and progenitor cells. Within the non-B/T cell compartment, CD14 and HLADR expression distinguished monocytes (CD14^+^HLADR^+^/^−^) and DCs (CD14^−^HLADR^+^) from granulocytes and NK cells (CD14^−^HLADR^−^) (Figure S1(G1–2)). Among CD14^−^HLADR^−^ cells, CD123^+^ cells were identified as basophils, and CD56^+^ cells as NK cells; NK cells could be further subclassified based on the expression levels of CD56 and CD16. Within the CD14^−^HLADR^+^ DC population, CD11c and CD123 were used to differentiate plasmacytoid DCs from monocytoid DCs (Figure S1(G8)). Among CD14^+^ monocytes, three subsets were identified: classical (HLADR^+^CD16^−^), non-classical (HLADR^+^CD16^+^), and an HLADR^−^CD16^−^ subpopulation containing myeloid-derived suppressor cells (MDSCs) (Figure S1(G3–5,G9–10)).

T cells were initially isolated based on the expression of CD4 and CD8 (Figure S1(C1)). Using surface markers and chemoattractant receptors, we identified activated T cells, regulatory T cells, memory subsets, and helper T (Th) populations (Figure S1(C2–3,C7)). Activation status was assessed via HLADR and CD38 expression, with activated CD4^+^ T cells defined as CD38^+^HLADR^+^ and CD38^+^HLADR^−^ subsets (Figure S1(C8)). Based on CD45RA and CCR7 expression, CD4^+^ and CD8^+^ T cells were categorized into naïve (CD45RA^+^CCR7^+^), effector (Teff; CD45RA^+^CCR7^−^), effector memory (Tem; CD45RA^−^CCR7^−^), and central memory (Tcm; CD45RA^−^CCR7^+^) subsets (Figure S1C).

In CD4^+^ memory T cells (CD3^+^CD4^+^CD45RA^−^), the expression of chemoattractant receptors distinguishes distinct T helper (Th) cell subsets. CCR10^−^CXCR5^+^ cells encompass follicular helper T (Tfh) cells (Figure S1(C3)). Among CCR10^−^CXCR5^−^ cells, the CCR6^+^CCR4^−^ phenotype defines Th9 cells (Figure S1(C4)). Further stratification based on CCR6, CCR4, CXCR3, and CCR10 expression enables the identification of Th1 (CXCR3^+^), Th2 (CXCR3^−^CCR10^−^), ThGM-CSF (CXCR3^−^CCR10^+^), Th17 (CCR6^+^CCR4^+^CXCR3^+^/^−^CCR10^−^), and Th22 (CCR6^+^CCR4^+^CXCR3^−^CCR10^+^) cells (Figure S1(C4–5,C10)).

B-cell subsets are defined by combinations of multiple markers. Based on CD38 and CD45RA expression: cells with high CD38 expression and low CD45RA expression are in a transitional or activated state; those with low CD38 expression and high CD45RA expression are in a naive state; and those with low CD38 expression and low CD45RA expression are in a mature or resting memory state. According to CD38 and CD25: CD38^+^CD25^+^ cells are activated, responsive to IL-2, and potentially exhibit regulatory functions; CD38^−^CD25^+^ cells are in a state of low-level activation; and CD38^+^CD25^low^ cells are in a resting or non-activated state. Based on CD45RA and HLADR: HLADR^+^CD45RA^high^ cells represent an activated naive state; HLADR^+^CD45RA^low^ cells represent an activated memory state; and HLADR^−^CD45RA^low^ cells represent a resting memory state.

### 2.5. Statistical Analysis

Continuous demographic and immune variables are presented as medians with interquartile ranges (IQR). Given that immune subset frequencies may not strictly adhere to a normal distribution, group comparisons were performed using the non-parametric Mann–Whitney U test. Statistical significance was initially evaluated at a two-tailed nominal *p* < 0.05. Given the exploratory nature of this highly multiplexed study involving 74 immune cell populations and 10 clinical groups, *p*-values were subsequently adjusted within each immune lineage compartment using the Benjamini–Hochberg false discovery rate (FDR) method. Specifically, FDR correction was applied independently within T-cell, B-cell, monocyte, NK cell, and dendritic cell subsets, rather than across all 74 populations globally. This compartment-specific approach effectively controls for multiple comparisons within biologically coherent groups while preserving sensitivity for lineage-specific signals.

To avoid over-interpretation, a strict three-tier hierarchy of evidence was established: (1) findings with an adjusted q < 0.10 were considered statistically robust discoveries. (2) Findings with a nominal *p* < 0.05 but q ≥ 0.10 were explicitly classified as hypothesis-generating exploratory trends requiring independent validation. (3) Findings with *p* ≥ 0.05 were interpreted as negative. This framework ensures that only the most robust, FDR-validated findings support our primary conclusions, while nominally significant results are transparently flagged. Exact *p*-values and q-values are reported throughout to facilitate transparent interpretation. Furthermore, secondary analyses (composite indices, severity gradients, and discriminant AUCs) were employed to strengthen conclusions beyond single-marker analyses. All statistical analyses were performed using Python SciPy (version 1.11), statsmodels (version 0.14), and scikit-learn (version 1.3). Data visualizations were generated using Python matplotlib, displaying median trends with interquartile range shading across disease groups.

### 2.6. Post Hoc Statistical Power Analysis

Given the exploratory design and modest sample sizes in rare phenotypic subgroups, we conducted post hoc power analysis for key immunological findings. Power was estimated for Mann–Whitney U tests using the normal approximation method based on observed Cohen’s d effect sizes. Effect sizes were calculated as absolute median difference divided by typical within-group standard deviation for each marker category (monocyte SD ≈ 5%, T-cell SD ≈ 8%, B-cell SD ≈ 4%, NK/DC SD ≈ 3%). Findings were classified by post hoc power: HIGH (≥80%), MARGINAL (50–79%), LOW (20–49%), or CRITICAL (<20%). The CD16^−^ classical monocyte expansion (Cohen’s d ≈ 8.0, power > 99%), CD8^+^ Teff collapse (d ≈ 1.84, power = 97%), and B-cell alterations in SMPP-GC (d = 6.3–7.2, power > 99%) are robust. By contrast, Treg depletion (Δ = −0.31%, d ≈ 0.06, power = 5%) and Th17 depletion (Δ = −0.48%, d ≈ 0.10, power = 6%) are CRITICALLY underpowered; their biological significance is uncertain despite nominal statistical significance. CD56^−^CD11C^+^ monocyte depletion in SMPP&BO (*n* = 3) should be considered preliminary regardless of *p*-value magnitude.

### 2.7. Secondary Analysis and Composite Immune Indices

Five composite immune indices were constructed to evaluate the coordinated dysregulation of entire immune axes. To capture the bidirectional shifts within specific immune compartments, indices were formulated using physiological ratios of the respective raw subset percentages [17]. Specifically, the indices were calculated as follows:

(i.) CD8^+^ T-cell Exhaustion Index = [(CD8^+^ Naïve) + (CD8^+^ Tcm)]/[(CD8^+^ Teff) + (CD8^+^ Tem3)], reflecting the numerator accumulation of undifferentiated and early-memory cells relative to denominator depletion of terminal effector populations.
(ii.) Adaptive Exhaustion Score = 100/(Treg^+^ Th17^+^ CD4^+^ Teff^+^ CD8^+^ Teff), reflecting the parallel loss of effector and regulatory T cells; higher values indicate more severe adaptive immune exhaustion.
(iii.) B-cell Maturation Arrest Index = [(HLADR^+^CD45RA^high^) + (CD38^−^CD45RA^+^)]/(CD38^+^CD25^low^), quantifying the blockade at early activation checkpoints; the numerator captures activated and naive B-cell accumulation while the denominator reflects transitional B-cell depletion.
(iv.) Innate-Adaptive Ratio = Total Monocytes/Total T cells in PBMCs, computed as a raw ratio reflecting the global shift from adaptive to innate immune dominance.

Severity-gradient analysis employed Spearman rank correlation against an ordinal severity scale (mild = 1, moderate = 2, severe uncomplicated = 3, Grade A = 3.5, Grade B = 4, Grade C = 4.5). Multi-marker discriminant power was quantified by the area under the receiver operating characteristic curve (AUC).

## 3. Results

### 3.1. Severity-Associated PBMC Architecture Restructuring

Analysis of major immune populations within PBMCs revealed selective alterations associated with disease severity and imaging subtypes (Figure S2). CD3^+^ T cells constituted the predominant PBMC subset across all groups (40–67% median), with MNMPP-N and MP-HSP groups showing higher proportions than severe MPP subtypes. CD20^+^ B cells demonstrated significant variation, with SMPP-GA patients exhibiting elevated proportions compared to SMPP-GC (*p* = 0.048, Delta = +2.6%) and MMPP-N (*p* = 0.016, Delta = +5.2%), suggesting enhanced B-cell involvement in mucous plug pathology. Dendritic cells remained relatively stable across groups.

Low-density granulocytes showed notable elevation in SMPP&BO patients (median ~27%) compared to other groups (~10–20%), consistent with neutrophil-predominant inflammation in obliterative airway disease. Natural killer cells demonstrated a divergent pattern between pathological grades, with SMPP-GB patients showing significantly reduced NK proportions compared to SMPP-GC (*p* = 0.047, Delta = −4.1%). Most strikingly, total monocytes in PBMCs were profoundly elevated in SMPP-GB (mucosal necrosis) compared to both SMPP-GC (*p* = 0.045, Delta = +12.2%) and MMPP-N (*p* = 0.044, Delta = +13.2%), suggesting that Grade B may represent an inflammatory monocyte-dominant phenotype. These exploratory PBMC-level findings suggest a potential shift in the immune population architecture during severe MPP.

### 3.2. T-Cell Activation States Define Complication-Specific Signatures

Comprehensive analysis of T-cell general and activation markers revealed potential compartment-specific alterations in severe complicated MPP (Figure S3). The CD4^+^ T-cell proportion within CD3^+^ T cells remained relatively stable across groups (median 50–64%), while CD8^+^ T cells showed modest elevation in SMPP&BO patients (~41%) compared to SMPP-N (~33%), suggesting CD8-biased responses in obliterative disease. The most prominent activation marker alteration involved CD38 expression on CD4^+^ T cells: SMPP&BO patients exhibited markedly elevated CD38^+^CD4^+^ proportions compared to SMPP-N (*p* = 0.017, Delta = +13.8%), while SMPP&PE patients showed decreased CD38^+^CD4^+^ compared to MMPP-N (*p* = 0.041, Delta = −14.3%), and the aggregated severe group (SMPP-ALL, combining SMPP&BO, SMPP&PE, SMPP-N, SMPP-GA, SMPP-GB, and SMPP-GC) demonstrated significant reduction versus MMPP-N (*p* = 0.013, Delta = −12.7%), suggesting activation exhaustion in PE but persistent activation in BO. These findings should be interpreted as preliminary due to the extremely limited sample size (*n* = 3); replication in larger BO-associated MPP cohorts is required before clinical application.

Double-negative T cells (CD8^−^CD4^−^), predominantly representing gamma-delta T cells and invariant NK-T cells, showed consistent depletion in severe complicated phenotypes, which might hint at a potential association between innate-like T-cell variations and necrotic or pleural complications.

### 3.3. CD4^+^ T-Cell Subpopulations Exhibit Complication-Dependent Memory Redistribution

**Figure 1:**
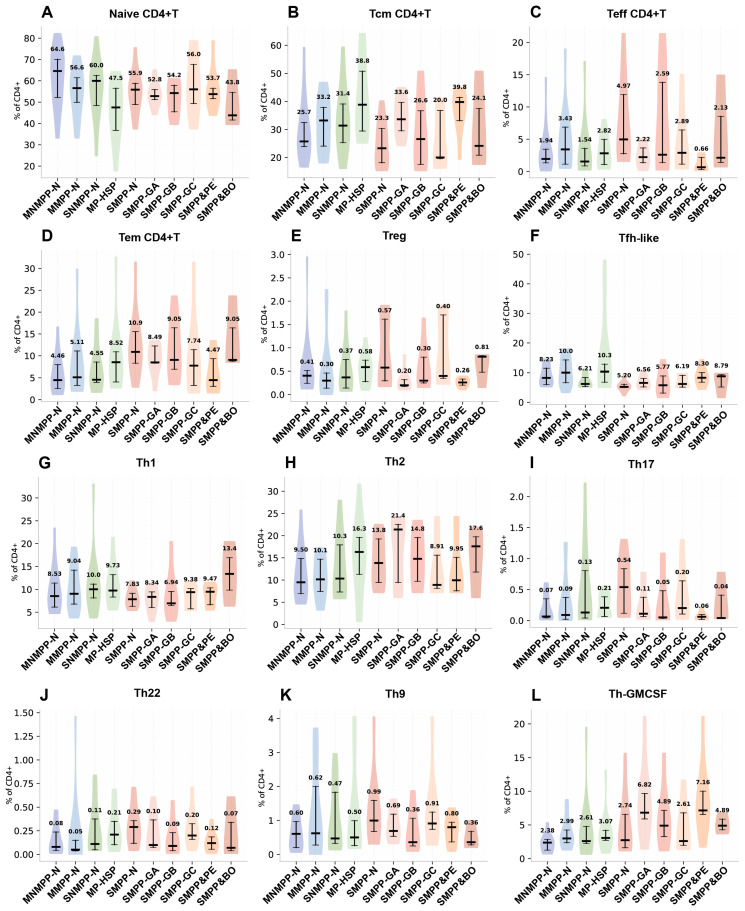
CD4^+^ T-cell subpopulation and Th functional subset distributions in MPP and NMPP subgroups. (**A**–**F**) Proportions of major cell populations in CD4^+^T cell (Naïve CD4^+^T cells, Tcm CD4^+^T cells, Teff CD4^+^T cells, Tem CD4^+^T cells, Treg cells, Tfh-like CD4^+^T cells). (**G**–**L**) T helper cell subsets of CD4^+^T cells (Th1, Th2, Th17, Th22, Th9, Th-GMCSF). Ten disease groups: MNMPP-N (*n* = 14), MMPP-N (*n* = 12), SNMPP-N (*n* = 11), MP-HSP (*n* = 10), SMPP-N (*n* = 10), SMPP-GA (*n* = 6), SMPP-GB (*n* = 5), SMPP-GC (*n* = 7), SMPP&PE (*n* = 8), SMPP&BO (*n* = 3). Data is presented as median with interquartile range (IQR). Statistical significance between groups was determined using the Mann–Whitney U test. Exact *p*-values are reported in the text.

Detailed profiling of CD4^+^ T-cell subpopulations revealed distinctive memory and functional subset alterations mapping closely to specific clinical complications (Figure 1). SMPP&PE patients exhibited pronounced CD4^+^ memory redistribution: central memory T cells (Tcm) were significantly elevated versus SMPP-N (*p* = 0.009, Delta = +16.5%), while effector T cells (Teff) decreased (*p* = 0.012, Delta = −4.3%), effector memory T cells (Tem) reduced (*p* = 0.045, Delta = −6.4%), and T follicular helper-like cells showed compensatory elevation (*p* = 0.035, Delta = +3.1%).

Regulatory and immunomodulatory CD4^+^ subsets were significantly depleted in pleural effusion. Treg proportions showed modest quantitative reduction in SMPP&PE versus SMPP-N (*p* = 0.022, Delta = −0.31%, post hoc power = 5%), while Th17 cells showed similar small-magnitude depletion (*p* = 0.008, Delta = −0.48%, post hoc power = 6%); given their CRITICALLY low statistical power and uncertain biological significance, these findings were interpreted with caution (Figure 1). ThGM-CSF-producing cells showed a reciprocal pattern, with increased proportions in SMPP&PE versus MMPP-N (*p* = 0.039, Delta = +4.2%) and in SMPP-ALL versus MMPP-N (*p* = 0.034, Delta = +3.8%), suggesting a potential association with myeloid activation in severe inflammatory disease. Th1 cells were selectively decreased in SMPP-GA versus MMPP-N (*p* = 0.047, Delta = −3.7%), while Th2, Th9, and Th22 subsets showed no significant differences.

### 3.4. CD8^+^ T-Cell Compartment Exhibits Progressive Exhaustion Across Severe Disease

**Figure 2:**
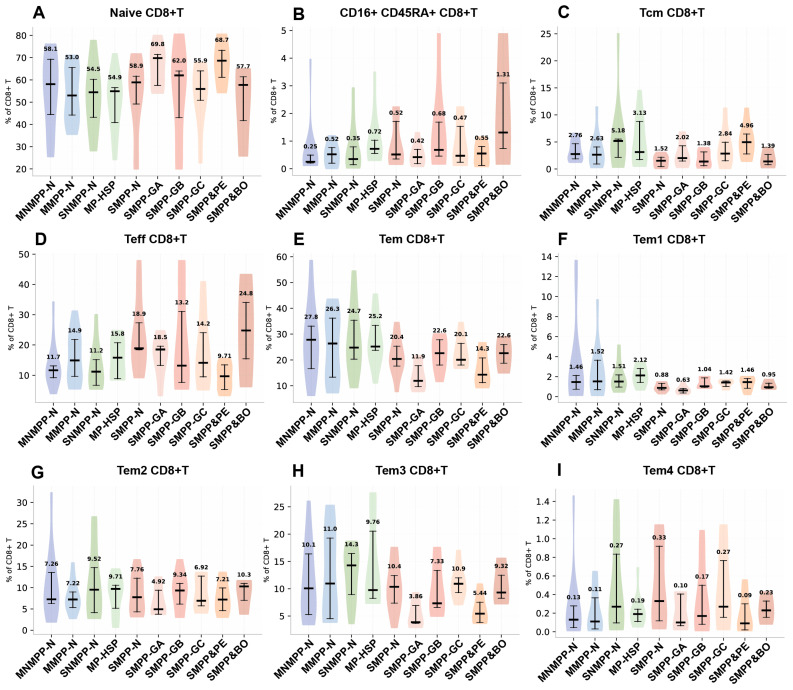
CD8^+^ T-cell subpopulation and Tem CD8^+^T cells subset distributions in MPP and NMPP subgroups. (**A**–**E**) Proportions of major cell populations in CD8^+^T cell (Naïve CD8^+^T cells, CD16^+^ CD45RA^+^ CD8^+^T cells, Tcm CD8^+^T cells, Teff CD8^+^T cells, Tem CD8^+^T cells). (**F**–**I**) Tem cell subsets of CD8^+^T cells (Tem1 CD8^+^T cells, Tem2 CD8^+^T cells, Tem3 CD8^+^T cells, Tem4 CD8^+^T cells). Ten disease groups: MNMPP-N (*n* = 14), MMPP-N (*n* = 12), SNMPP-N (*n* = 11), MP-HSP (*n* = 10), SMPP-N (*n* = 10), SMPP-GA (*n* = 6), SMPP-GB (*n* = 5), SMPP-GC (*n* = 7), SMPP&PE (*n* = 8), SMPP&BO (*n* = 3). Data is presented as median with interquartile range (IQR). Statistical significance between groups was determined using the Mann–Whitney U test. Exact *p*-values are reported in the text.

The CD8^+^ T-cell compartment demonstrated the most notable severity-associated alterations (Figure 2). SMPP&PE patients exhibited a pronounced naive-effector imbalance: CD8^+^ naive T cells were significantly elevated (*p* = 0.035, Delta = +9.7% vs. SMPP-N; *p* = 0.015, Delta = +15.6% vs. MMPP-N), while effector T cells (Teff) showed marked reduction (*p* = 0.008, Delta = −9.2% vs. SMPP-N; *p* = 0.039, Delta = −5.2% vs. MMPP-N).

Terminal effector memory subset 3 (CD8^+^Tem3) emerged as a notable potential indicator of the severity gradient. SMPP-GA patients showed significantly higher Tem3 than SMPP-GB (*p* = 0.030) and SMPP-GC (*p* = 0.003, Delta = −7.1%), with SMPP-GB also exceeding SMPP-GC (*p* = 0.039), indicating a trend of progressive Tem3 decline from Grade A through C. Against MMPP-N controls, SMPP-GA showed higher Tem3 (*p* = 0.012), while no difference existed between SMPP-GC and MMPP-N, suggesting Tem3 depletion as a potential indicator of maximal severity. Total CD8^+^ Tem showed concordant patterns (GA vs. GC: *p* = 0.007, Delta = −9.0%; GA vs. MMPP-N: *p* = 0.010, Delta = −15.3%). CD8^+^ central memory (Tcm) cells were elevated in SMPP&PE versus SMPP-N (*p* = 0.016, Delta = +3.4%), complementing the blocked differentiation pattern.

### 3.5. B-Cell Maturation Signatures Map to Airway Pathology

**Figure 3:**
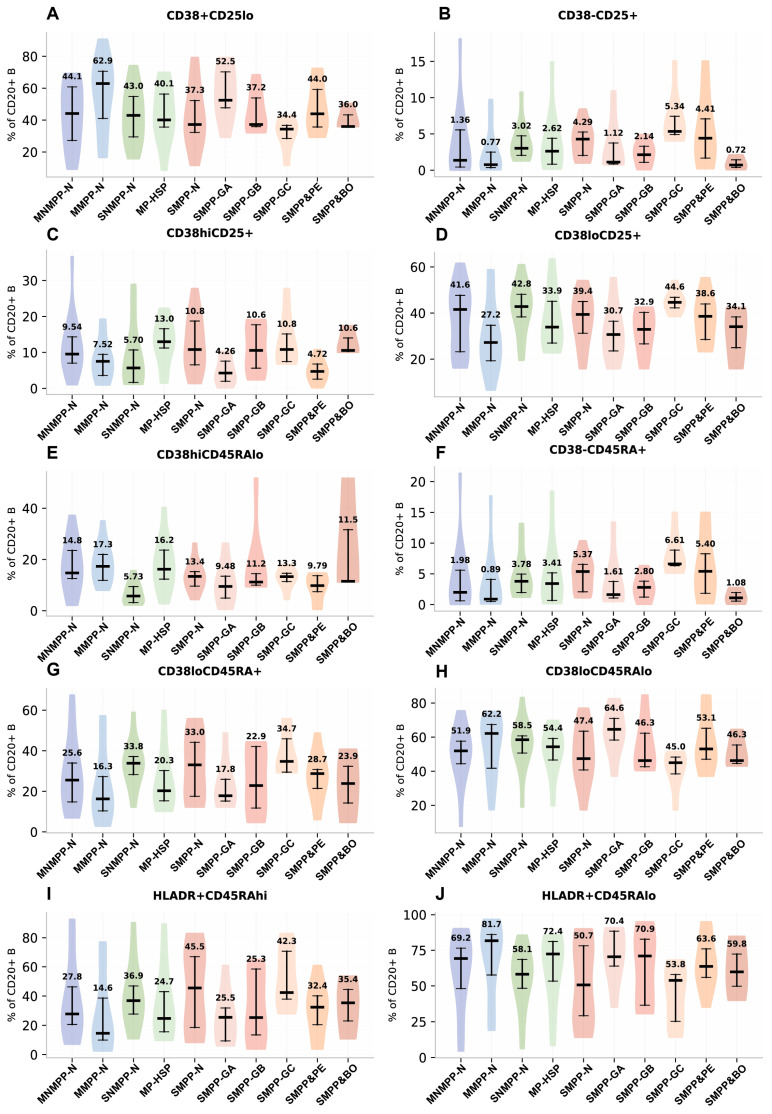
B-cell subpopulation in MPP and NMPP subgroups. (**A**–**D**) B cell defined by CD38 and CD25 (CD38^+^ CD25^low^ B cells, CD38^−^CD25L^+^ B cells, CD38hi CD25^+^ B cells, CD38^low^ CD25^+^ B cells). (**E**–**H**) B-cell subsets defined by CD38 and CD45RA (CD38hi CD45RA^low^ B cells, CD38^−^CD45RA^+^ B cells, CD38^low^ CD45RA^+^ B cells, CD38^low^ CD45RA^low^ B cells). (**I**,**J**) B-cell subsets defined by HLADR and CD45RA (HLADR^+^CD45RAhi B cells, HLADR^+^ CD45RA^low^ B cells). Ten disease groups: MNMPP-N (*n* = 14), MMPP-N (*n* = 12), SNMPP-N (*n* = 11), MP-HSP (*n* = 10), SMPP-N (*n* = 10), SMPP-GA (*n* = 6), SMPP-GB (*n* = 5), SMPP-GC (*n* = 7), SMPP&PE (*n* = 8), SMPP&BO (*n* = 3). Data is presented as median with interquartile range (IQR). Statistical significance between groups was determined using the Mann–Whitney U test. Exact *p*-values are reported in the text.

Profiling of B-cell subpopulations revealed marked maturation and activation alterations that mapped specifically to imaging and bronchoscopic pathological grades (Figure 3). CD20^+^ B cells demonstrated significant variation across disease groups, with SMPP-GA (mucous plugs) patients exhibiting elevated proportions compared to SMPP-GC (diffuse bronchiolitis; *p* = 0.048, Delta = +2.6%) and MMPP-N (moderate MPP; *p* = 0.016, Delta = +5.2%), suggesting enhanced B-cell involvement in mucous plug pathology. By contrast, SMPP-GC showed the most distinctive B-cell maturation signature, characterized by coordinated transitional B-cell depletion and activated B-cell expansion.

Transitional B cells (CD38^+^CD25^low^), representing a critical developmental checkpoint between immature bone marrow emigrants and mature peripheral B cells, were significantly depleted in SMPP-GC compared to SMPP-GA (*p* = 0.034, Delta = −4.1%), suggesting a potential impairment or delay at early B-cell activation checkpoints. This depletion was accompanied by reciprocal expansion of activated B cells (HLADR^+^CD45RA^high^) in SMPP-GC versus SMPP-GA (*p* = 0.028, Delta = +3.1%), suggesting diversion of B-cell development away from transitional tolerance toward premature activation. Naive B cells (CD38^−^CD45RA^+^) showed corresponding accumulation in SMPP-GC, consistent with impaired maturation progression.

Germinal center B cells (CD38^high^CD45RA^low^), which are essential for affinity maturation and long-term humoral immunity, were notably depleted in both SMPP-GA and SMPP-PE groups compared to mild MPP, suggesting germinal center disruption in severe disease. This observation aligns with emerging evidence from severe respiratory infections, where loss of germinal center architecture impairs viral clearance and disease resolution. Notably, the HLADR^+^ phenotype switching in SMPP-GC indicates an alternative activation pathway distinct from the germinal center disruption seen in other severe phenotypes, potentially reflecting unique small airway inflammatory signals in diffuse bronchiolitis.

The B-cell Maturation Arrest Index [(HLADR^+^CD45RA^high^) + (CD38^−^CD45RA^+^)]/(CD38^+^CD25^low^) quantified this blockade and effectively discriminated SMPP-GC from SMPP-GA (*p* = 0.031, Cohen’s d = −1.43). For SMPP-GC versus mild MPP discrimination, the index achieved AUC = 0.80 (Figure S5). These exploratory findings suggest that B-cell maturation arrest may serve as a potential immunological signature in severe MPP, contrasting with the T-cell exhaustion and monocyte inflammatory patterns that characterize pleural effusion.

### 3.6. Innate Immune Dysregulation Defines Complication Signatures

**Figure 4:**
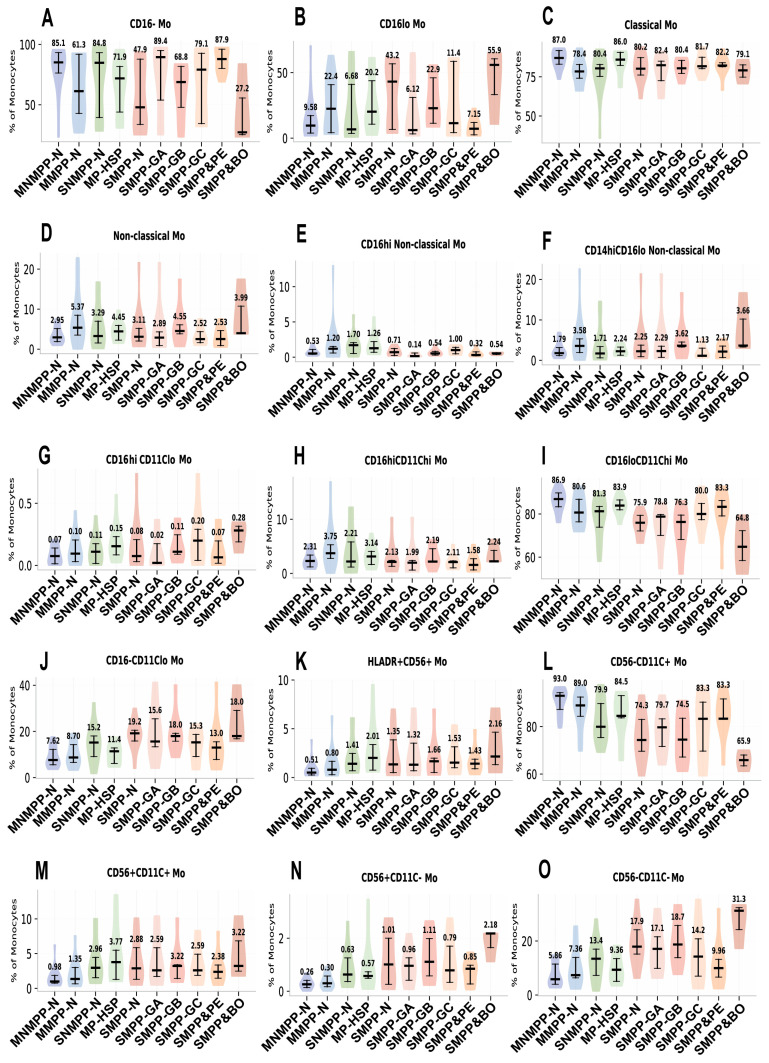
Monocyte subset distribution and activation states. (**A**,**B**) Monocytes were defined by CD16 (CD16^+^ Monocytes and CD16^+^ Monocytes). (**C**,**D**) Classical Monocytes and non-classical Monocytes subsets in Monocytes. (**E**,**F**) Non-classical Monocytes were defined by CD16 and CD14 (CD16hi non-classical Monocytes and CD14hi CD16^low^ non-classical Monocytes). (**G**–**J**) Monocytes was defined by CD16 and CD11C (CD16hi CD11C^low^ Monocytes, CD16hi CD11Chi Monocytes, CD16^low^ CD11Chi Monocytes, CD16^−^ CD11C^low^ Monocytes). (**K**) The proportion of HLADR^+^CD56^+^ Monocytes in Monocytes. (**L**–**O**) Monocytes were defined by CD56 and CD11C (CD56^−^CD11C^+^ Monocytes, CD56^+^CD11C^+^ Monocytes, CD56^+^CD11C^−^ Monocytes, CD56^−^CD11C^−^ Monocytes). Data is presented as median with interquartile range (IQR). Statistical significance between groups was determined using the Mann–Whitney U test. Exact *p*-values are reported in the text.

Preliminary data in the small BO cohort (*n* = 3) suggested a fundamentally different monocytic signature: CD56^−^CD11C^+^ monocytes were significantly depleted versus MMPP-N (*p* < 0.001, Delta = −23.1%), which might be hypothetically linked to the fibrotic airway remodeling process, though this requires validation. CD56^+^CD11C^−^ monocytes were elevated in SMPP-ALL versus MMPP-N (*p* = 0.003, Delta = +0.6%), representing a previously unrecognized severe disease-associated activation state (Figure 4).

Dendritic cell and NK cell populations showed selective alterations (Figure S4). CD11c^low^CD25^high^ plasmacytoid DCs were significantly elevated in SMPP-GA versus MMPP-N (*p* = 0.008, Delta = +13.8%) and in SMPP-ALL versus MMPP-N (*p* = 0.049, Delta = +12.5%). NK cells polarized functionally in severe disease: CD56^high^CD16^int^ cytokine-producing NK cells were elevated in SMPP-ALL versus MP-HSP (*p* = 0.003, Delta = +3.6%), while CD16^high^ cytotoxic NK cells showed a reciprocal decrease (*p* = 0.023, Delta = −9.1%).

### 3.7. Secondary Analysis I: Composite Immune Indices (Hypothesis-Generating)

To move beyond single-marker comparisons and capture coordinated immune axis dysregulation, we constructed four composite indices using physiological ratios of raw subset percentages (Methods 2.7). The CD8^+^ T-cell Exhaustion Index ((Naïve + Tcm)/(Teff + Tem3)) was significantly elevated in SMPP&PE versus MMPP-N (*p* = 0.003, Cohen’s d = 1.50), capturing the blocked differentiation from naive through effector to terminal memory stages. The Adaptive Exhaustion Score (100/(Treg^+^Th17^+^CD4^+^Teff^+^CD8^+^Teff)) demonstrated the strongest single-index discrimination for PE (AUC = 0.90 versus uncomplicated SMPP), quantifying the parallel loss of effector and regulatory T-cell function. The B-cell Maturation Arrest Index [(HLADR^+^CD45RA^high^) + (CD38^−^CD45RA^+^)]/(CD38^+^CD25^low^) discriminated SMPP-GC from SMPP-GA (*p* = 0.031, Cohen’s d = −1.43), reflecting the blockade at early B-cell activation checkpoints (Figure S5A).

The combination of Monocyte Inflammation Index with Adaptive Exhaustion Score may provide a dual-axis framework for simultaneously predicting PE risk and severity grade. These high AUC values underscore its strong discriminative potential within our cohort, though they remain exploratory and require validation in independent cohorts before any clinical application.

Although individual quantitative reductions in Treg and Th17 subsets lacked adequate statistical power (as noted in Section 2.4), they were retained in the denominator of the Adaptive Exhaustion Score to theoretically represent the global pool of adaptive regulatory and effector cells. The robust discriminatory power of this composite index (AUC = 0.90) is primarily driven by the significant reduction in CD8^+^ Teff cells. Although these high AUCs suggest strong internal discrimination, we acknowledge the risk of overfitting, and these indices currently serve as hypothesis generators rather than clinical biomarkers.

### 3.8. Secondary Analysis II: Innate-Adaptive Rebalancing

A central insight emerged from computing the Innate-Adaptive Ratio (monocytes/T cells in PBMCs): this ratio increased progressively and significantly with disease severity (Spearman rho = +0.40, *p* = 0.0002). Mild MPP patients showed ratios near 0.1, while severe SMPP-GB patients exceeded 0.5, indicating a fundamental shift from adaptive T-cell-dominated immunity toward innate monocyte-driven inflammation. This innate-adaptive rebalancing may represent a key inflammatory feature associated with complication-specific phenotypes (Figure S5B).

Severity-gradient analysis (ordinal scale: mild = 1, moderate = 2, severe uncomplicated = 3, Grade A = 3.5, Grade B = 4, Grade C = 4.5) confirmed the dominance of monocyte and innate markers in driving severity-associated immune changes. CD56^−^CD11C^+^ monocytes showed the strongest severity gradient (rho = −0.470, *p* = 0.0003), followed by activated CD4^+^ T cells (rho = +0.460), CD16^−^CD11c^low^ monocytes (rho = +0.458), and total CD3^+^ T cells (rho = −0.437). The heatmap visualizes coordinated restructuring across the top ten severity-gradient markers: as severity increases, T cells contract while monocyte subsets expand and B-cell maturation arrests.

### 3.9. Secondary Analysis III: Multi-Marker Discriminant Power

Area-under-curve analysis quantified discriminatory power for three key phenotype comparisons (Figure S5C). For PE versus uncomplicated SMPP, the Adaptive Exhaustion Score achieved AUC = 0.90, followed by the CD8 Exhaustion Index (AUC = 0.88), CD4^+^ Tcm (AUC = 0.86), and CD16- monocytes (AUC = 0.80). For BO versus mild MPP, CD56^−^CD11C^+^ monocyte depletion demonstrated strong discrimination (AUC = 0.97), though this estimate should be interpreted cautiously given the small BO cohort (*n* = 3). For SMPP-GC versus mild MPP, the B-cell Maturation Arrest Index reached AUC = 0.80. Notably, the composite physiological-ratio indices consistently outperformed or matched their constituent single markers, highlighting the potential analytical value of integrated immune-axis assessment over isolated population measurements.

The convergence of single-marker findings, composite index discrimination, and severity-gradient correlation provides a coherent picture of SMPP immunopathogenesis. No single marker alone captures the complexity of severe MPP; rather, coordinated dysregulation of entire immune axes—innate inflammation, adaptive exhaustion, and B-cell maturation—appears to be associated with distinct complication phenotypes.

## 4. Discussion

This study provides three levels of advancement over prior MPP immunology research. First, we establish the most comprehensive immune atlas of pediatric MPP to date, profiling 74 subpopulations across the full clinical spectrum. Second, we identify distinct immunological signatures that precisely map specific complications and imaging phenotypes. Third, our secondary analysis reveals a unifying pathophysiological principle: severe MPP is fundamentally characterized by an innate-adaptive immune rebalancing that shifts the systemic environment from T-cell-dominated adaptive immunity toward monocyte-driven innate inflammation.

### 4.1. The Innate-Adaptive Rebalancing Axis: A Unifying Framework

The central finding of this investigation is the identification of innate-adaptive rebalancing as the core pathophysiological axis in severe MPP. The Innate-Adaptive Ratio increased progressively with severity (Spearman rho = +0.40, *p* = 0.0002), and severity-gradient analysis confirmed that monocyte expansion and T-cell contraction are the dominant coordinated changes across the disease spectrum. This finding transcends the traditional paradigm of simply listing altered cell subsets and instead positions immune compartment rebalancing as the mechanistic driver of clinical heterogeneity.

Within this framework, specific complications emerge as divergent manifestations of the same underlying axis. Pleural effusion represents the extreme pole of innate inflammatory dominance: massive CD16- classical monocyte mobilization (Delta = 40%) drives exudative inflammation through TNF-alpha, IL-1beta, and GM-CSF production, while concurrent adaptive exhaustion (CD8^+^Teff collapse, Treg/Th17 depletion) removes regulatory constraints. Bronchiolitis obliterans, conversely, reflects not innate excess but innate deficit: CD56^−^CD11C^+^ monocyte depletion (Delta = −23%) impairs tissue repair and matrix remodeling, permitting aberrant fibrotic scarring. Diffuse bronchiolitis occupies an intermediate position with prominent B-cell maturation arrest.

### 4.2. Comparison with Prior Literature

Our findings both confirm and extend prior reports of immune dysregulation in MPP. The massive CD16- classical monocyte expansion in PE (Delta = 40%) is strongly supported by established parapneumonic effusion pathophysiology: parapneumonic effusions are characterized by accumulation of neutrophils and mononuclear phagocytes as an initial innate response, with CCL2 (the principal chemoattractant for CD14+ classical monocytes) significantly elevated in pleural effusion versus transudate. Our observation of 40% expansion quantitatively exceeds prior reports and positions classical monocyte mobilization as a therapeutic target in PE-associated SMPP [18].

The CD8^+^ Teff collapse in PE (Delta = −9.2%) is strongly supported by multiple independent lines of evidence. A recent single-cell transcriptome atlas of pediatric MPP BALF cells has identified exhausted CD8^+^ T cells as a potential cause of severe disease, with CD8^+^ exhaustion subclusters showing high PDCD1, LAG3, and HAVCR2 expression. Studies in malignant pleural effusion further confirm that the pleural microenvironment accelerates T-cell exhaustion, with CD8^+^ T cells showing the highest PD-1 and lowest granzyme B and IFN-gamma expression among all compartments. Our findings extend these observations by demonstrating that CD8^+^ terminal effector memory subset 3 shows progressive depletion across severity grades, identifying Tem3 as a potential indicator of severity [19].

The B-cell maturation arrest signature in diffuse bronchiolitis (CD38^+^CD25^low^ depletion, HLADR^+^CD45RA^high^ expansion) aligns with emerging evidence from severe respiratory infections. In COVID-19, loss of germinal centers and Bcl-6-expressing T follicular helper cells impair humoral immunity and disease resolution. Our observation of CD38^high^CD45RA^low^ germinal center B-cell depletion in SMPP-GA and SMPP-PE suggests a similar germinal center disruption mechanism in severe MPP, while the HLADR+ phenotype switching in SMPP-GC indicates an alternative activation pathway in diffuse small airway inflammation [18].

### 4.3. Implications for Biomarker Development and Clinical Translation

The composite immune indices developed in this study demonstrate strong theoretical discriminative potential within our cohort: the Adaptive Exhaustion Score (AUC = 0.90 for PE prediction) and Monocyte Inflammation Index (Cohen’s d = 1.37). The combination of these indices with CD8^+^Tem3 as a severity gradient marker creates a dual-axis framework: the Innate-Adaptive Ratio indicates overall severity trajectory, while complication-specific signatures (CD16^−^ expansion for PE, CD56^−^CD11C^+^ depletion for BO, B-cell arrest for diffuse bronchiolitis) may correlate with specific complication risk. These findings align with the demonstrated clinical utility of composite inflammatory indices such as the systemic immune-inflammation index (SII) for severe MPP prediction [20]. These composite indices are presented as hypothesis-generating tools requiring independent internal or external validation before clinical application. The high AUCs achieved on the derivation dataset likely reflect some degree of overfitting, and the clinical utility of these indices should be confirmed in prospective, multi-center cohorts. They should not be interpreted as validated biomarkers ready for clinical stratification.

From a therapeutic perspective, these findings suggest that uniform immunosuppression may be suboptimal for all SMPP patients. PE-driven cases with high monocyte inflammation may benefit from early anti-cytokine or corticosteroid intervention targeting the exudative cascade, while BO-risk cases with CD56^−^CD11C^+^ depletion might respond better to strategies promoting tissue-reparative monocyte function or antifibrotic agents. The identification of GM-CSF as a potential driver of monocyte-mediated inflammation (via ThGM-CSF elevation in PE) raises the possibility of GM-CSF-targeted therapy, an approach already under investigation in other hyperinflammatory pneumonias. This precision immunomodulation approach, guided by peripheral immune profiling, represents a promising direction for future clinical trials, as underscored by recent calls for immune-stratified therapeutic strategies in the context of rising macrolide resistance [21]. It is important to emphasize that these composite indices and stratification frameworks represent proof-of-concept findings derived from a single-center cohort. Their clinical utility requires prospective validation in independent, multi-center studies with predefined endpoints. The AUC thresholds reported here likely include some degree of overfitting and should be interpreted as upper-bound estimates of true discriminant performance.

### 4.4. Limitations

This study has several limitations. First, the cross-sectional design precludes the determination of temporal immune dynamics during disease progression and convalescence. Second, the sample sizes for certain rare complication subgroups (particularly SMPP&BO, *n* = 3) are inherently limited, which constrains statistical power. Consequently, non-significant findings in these smaller subsets should be interpreted with caution rather than as definitive proof of equivalence. Although preliminary findings in the BO cohort revealed a striking depletion of CD56^−^CD11C^+^ monocytes (Delta = −23.1%), this profound dysregulation represents an exploratory, hypothesis-generating trend that necessitates rigorous validation, rather than a definitive clinical biomarker. Third, given the exploratory nature of this highly multiplexed immunophenotyping study, the Benjamini–Hochberg false discovery rate (FDR) method was applied independently within each immune lineage compartment. While this approach effectively controls multiple comparisons within biologically coherent groups, some Type I error inflation may remain across the entire 74 population landscape. Fourth, crucially, the composite metrics constructed in this study (e.g., the Adaptive Exhaustion Score and B-cell Maturation Arrest Index) were derived and evaluated within the same dataset. We explicitly acknowledge the high risk of statistical overfitting and the inherently optimistic nature of the reported AUC values. Therefore, these composite scores are strictly intended as heuristic models to summarize multidimensional phenotypic shifts, and they are not yet candidate tools for clinical stratification. Finally, our cohort was recruited from a single center, and functional validation of phenotypic alterations (e.g., cytokine secretion assays) was not performed. Furthermore, age differences between groups (MP-HSP median 11.4 years vs. NMPP median 5.9 years and MPP median 7.3 years) may confound immune comparisons. Although sensitivity analyses restricting the cohort to 5–10 years yielded directionally concordant findings, age-adjusted general linear models in larger cohorts should be pursued. Future multi-center prospective studies, ideally integrating multi-omics approaches [22], are essential to independently validate these exploratory immune profiles and resolve the outstanding mechanistic controversies surrounding immune dysregulation in MPP.

## 5. Conclusions

High-dimensional immune profiling reveals that severe pediatric MPP is driven by a core innate-adaptive rebalancing axis, with specific complications representing divergent manifestations of this central mechanism. Pleural effusion is characterized by extreme innate inflammatory dominance with adaptive exhaustion (CD16- monocyte expansion + CD8^+^Teff collapse, AUC = 0.90), bronchiolitis obliterans by preliminary evidence of tissue-reparative monocyte deficit (CD56^−^CD11C^+^ depletion, AUC = 0.97, *n* = 3, requiring validation), and diffuse bronchiolitis by B-cell maturation arrest (HLADR^+^CD45RA^high^ expansion CD38^+^CD25^low^ depletion). Composite immune indices show promise in differentiating specific phenotypes, serving as a hypothesis-generating framework for future prospective validation and targeted immunomodulatory intervention.

## Figures and Tables

**Table 1:** (**a**) Demographic and clinical characteristics of MPP and NMPP groups. (**b**) Demographic and clinical characteristics of SMPP subgroups.

**(a)**							
**Group**	**Number**	**Age (Years) Median [IQR]**	**Sex (M/F)**	**Group**	**Number**	**Age (Years) Median [IQR]**	**Sex (M/F)**
MPP	64	7.3 [5.7–8.4]	37/27	MMPP-N	12	7.1 [5.4–8.6]	7/5
				SMPP	42	7.3 [5.7–8.6]	24/18
				MP-HSP	10	11.4 [9.6–11.9]	6/4
NMPP	25	5.9 [4.1–8.6]	9/16	SNMPP-N	11	6.3 [4.3–7.9]	3/8
				MNMPP-N	14	5.3 [4.0–5.3]	6/8
**(b)**							
**Group**		**Number**	**Age (Years) Median [IQR]**			**Sex (M/F)**	
SMPP-N		10	7.0 [5.9–7.7]			5/5	
SMPP&BO		3	7.4 [5.4–8.10			2/1	
SMPP&PE		8	7.1 [6.5–7.6]			5/3	
SMPP-GA		7	7.3 [6.7–7.9]			5/2	
SMPP-GB		7	7.3 [6.7–8.2]			3/4	
SMPP-GC		7	7.2 [6.5–7.8]			4/3	

(a,b): Data presented as median [IQR] or *n* (%). SMPP subgroups: by bronchoscopy grade (GA-mucous plugs *n* = 6, GB-mucosal necrosis *n* = 5, GC-diffuse bronchiolitis *n* = 7); note these 18 phenotyped patients represent a bronchoscopically characterized subset of the total 21 SMPP patients; by complication (N-uncomplicated *n* = 10, PE-pleural effusion *n* = 8, BO-bronchiolitis obliterans *n* = 3). MPP = *Mycoplasma pneumoniae* pneumonia; HSP = Henoch-Schonlein purpura; GA/B/C = bronchoscopic severity grades; N = no complication; PE = pleural effusion; BO = bronchiolitis obliterans.

## Data Availability

The original flow cytometry data (FCS files) and datasets generated during the current study will be available in the FlowRepository repository upon acceptance (accession number to be assigned). De-identified clinical datasets and additional statistical analysis codes are available from the corresponding author upon reasonable request.

## Supplementary Materials

The following supporting information can be downloaded at: https://www.mdpi.com/article/10.3390/diagnostics16152429/s1.

## Abbreviations

MP	*Mycoplasma pneumoniae*
CAP	Community-acquired pneumonia
MPP	*Mycoplasma pneumoniae* pneumonia
SMPP	Severe *Mycoplasma pneumoniae* pneumonia
PE	Pleural effusion
BO	Bronchiolitis obliterans
NK cells	Natural killer cells
COVID-19	Coronavirus disease 2019
CD4^+^	Cluster of differentiation 4
CD8^+^	Cluster of differentiation 8
FBS	Fetal bovine serum
PBS	Phosphate-buffered saline
FcR	Fc receptor
MACS	Magnetic-activated cell sorting
EDTA	Ethylenediaminetetraacetic acid
BSA	Bovine serum albumin
FSC	Forward scatter
SSC	Side scatter
PBMCs	Peripheral blood mononuclear cells
DCs	Dendritic cells
MDSCs	Myeloid-derived suppressor cells
HLA-DR	Human leukocyte antigen—DR isotype
CD	Cluster of differentiation
Th	T helper cell
Tfh	T follicular helper cell
Th9	T helper 9 cell
Th1	T helper 1 cell
Th2	T helper 2 cell
Th17	T helper 17 cell
Th22	T helper 22 cell
Teff	Effector T cell
Tem	Effector memory T cell
Tcm	Central memory T cell
GM-CSF	Granulocyte-macrophage colony-stimulating factor
IL-2	Interleukin-2
CCR	C-C chemokine receptor
CXCR	C-X-C chemokine receptor
