# Supplementary material for: High-Dimensional Immune Profiling Reveals Innate-Adaptive Rebalancing and Subpopulation Trajectories in Pediatric Severe Mycoplasma pneumoniae Pneumonia
Source: Diagnostics (Basel). 2026 Jul 31;16(15):2429. doi: 10.3390/diagnostics16152429 (PMC13465347; doi:10.3390/diagnostics16152429)
Supplement: Supplementary file 1 [file diagnostics-16-02429-s001.zip › diagnostics-4393350-supplementary.pdf]

Supplementary Materials

（1）Supplementary Material S1

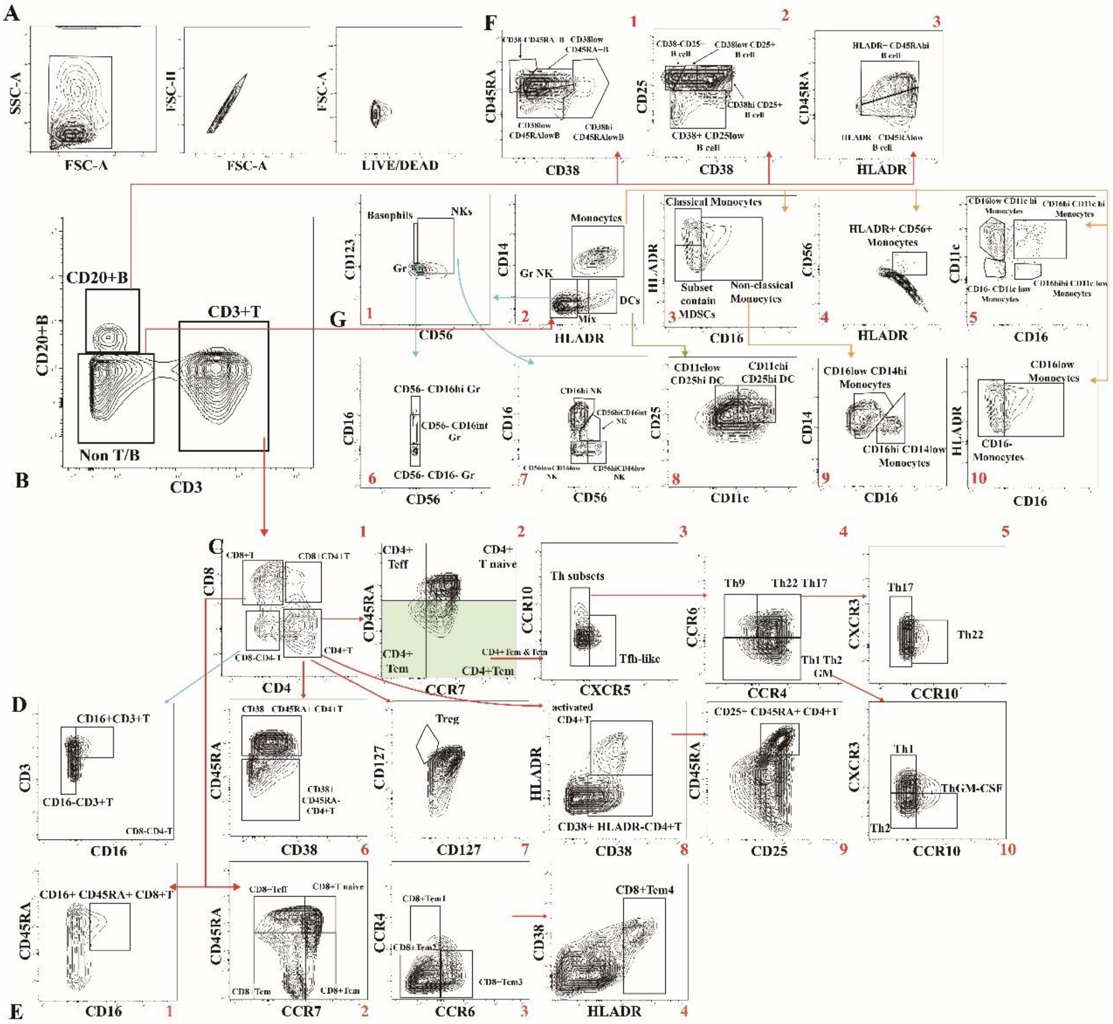

Supplementary Figure S1. Flow Cytometry Clustering Plots

Clustering analysis of peripheral blood mononuclear cell (PBMC) subsets using high-dimensional multicolor flow cytometry in 160 pediatric patients (64 MPP, 25 NMPP).

(2) Supplementary Material S2

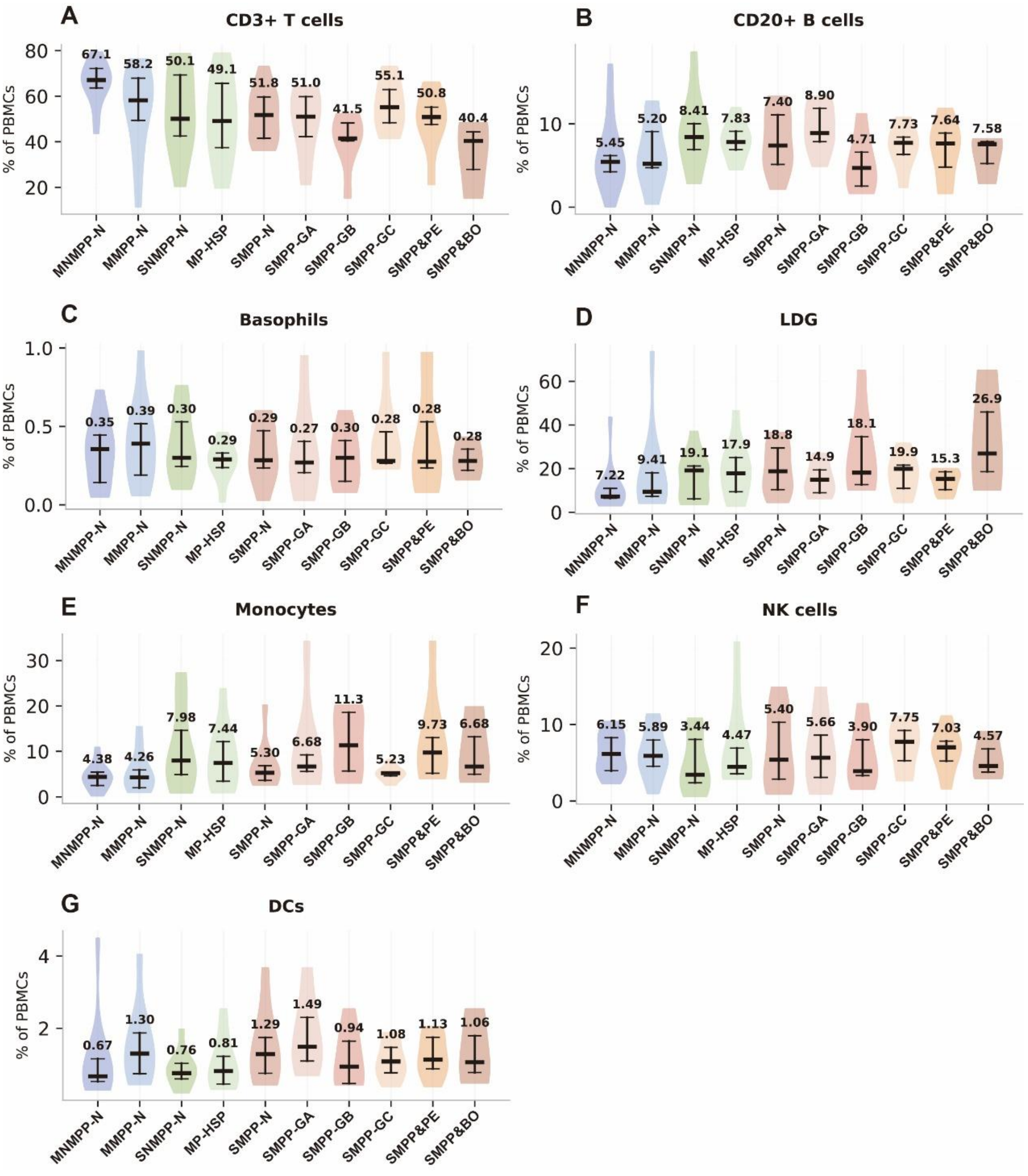

Supplementary Figure S2. PBMC major immune lineage distributions across ten MPP disease groups. Violin plots with embedded median (line) and IQR (box).

(3) Supplementary Material S3

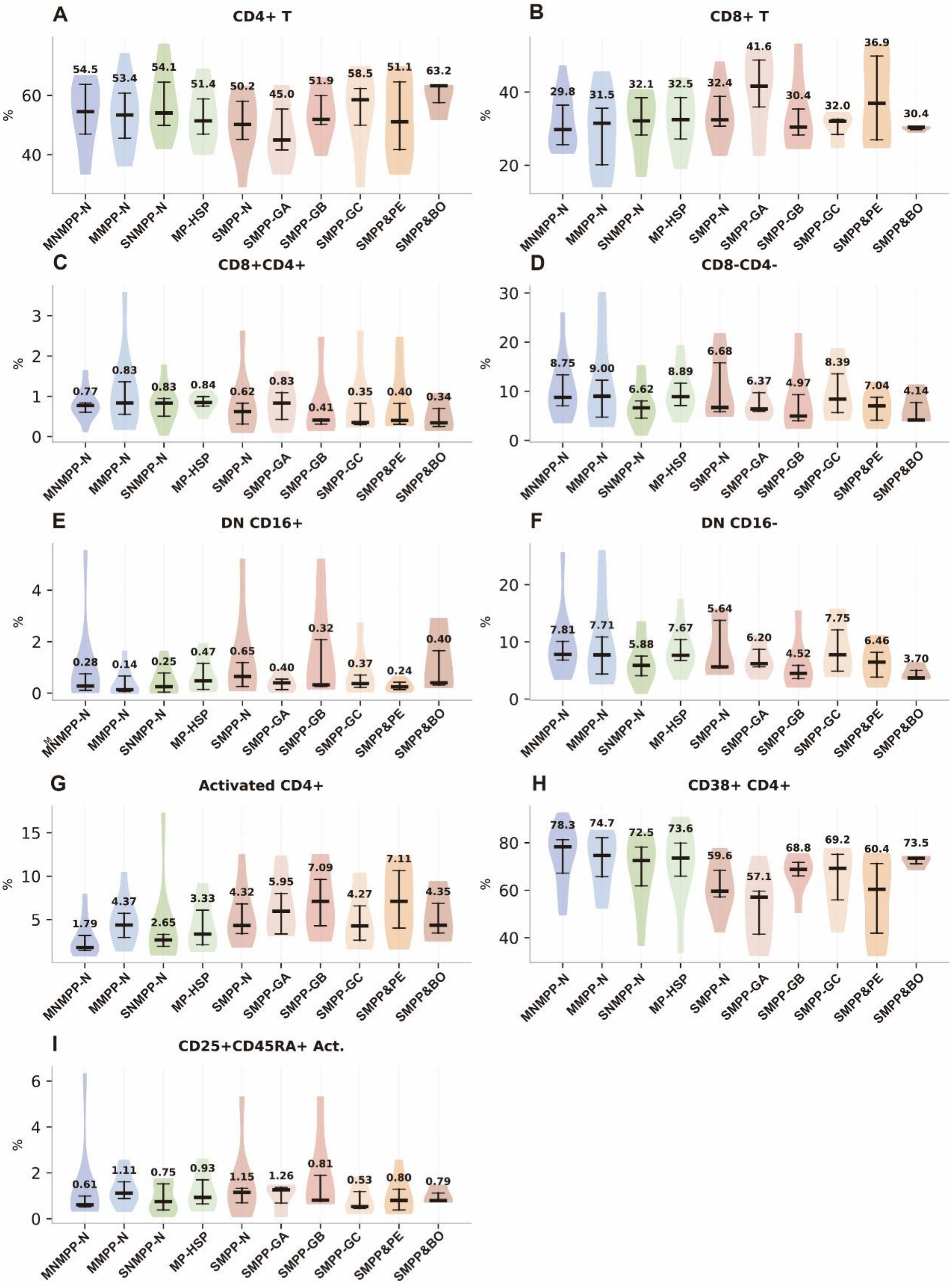

Supplementary Figure S3. T-cell composition and activation marker distributions across MPP disease groups.

(4) Supplementary Material S4

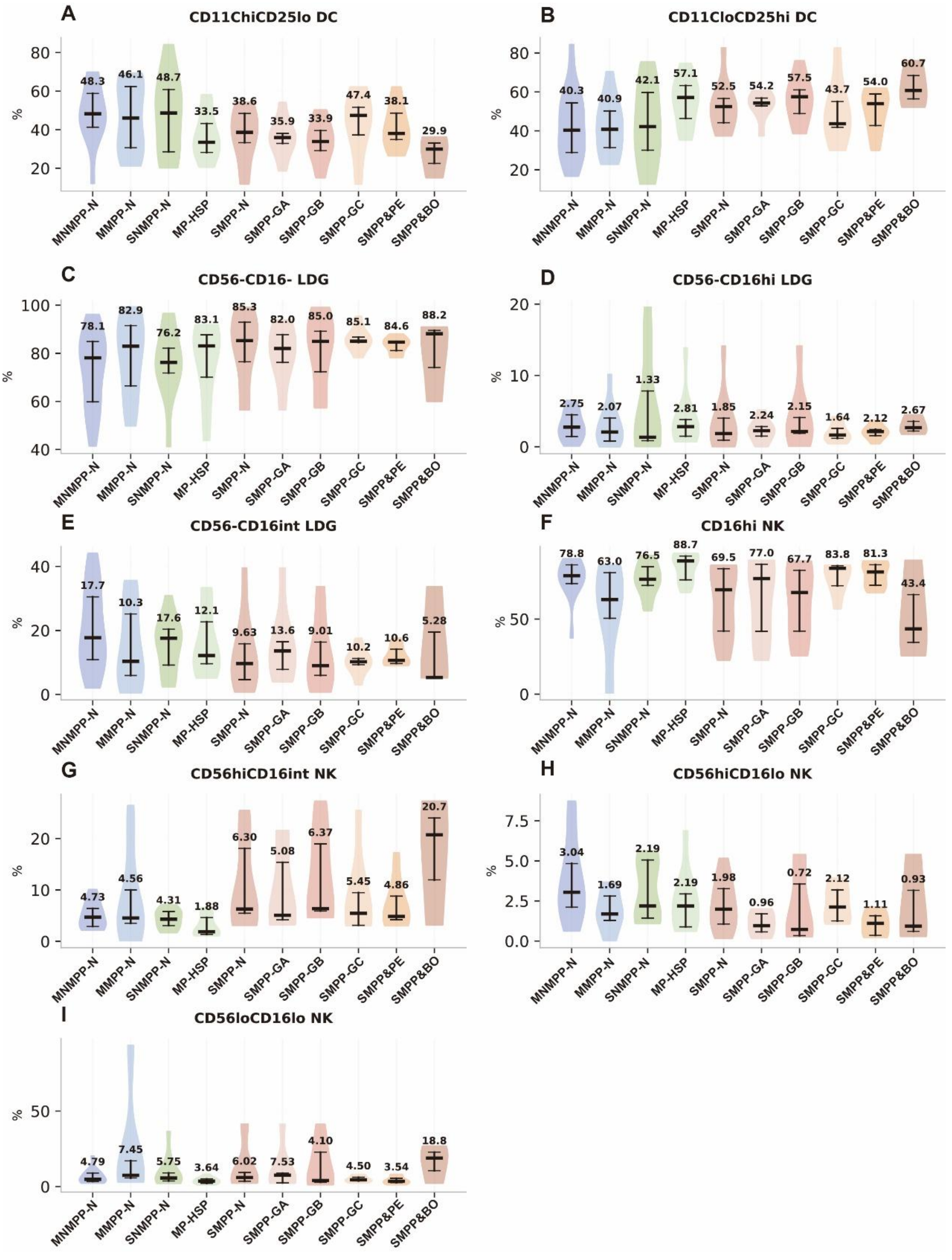

Supplementary Figure S4. Dendritic cells, NK cells, and granulocyte distributions.

(5) Supplementary Material S5

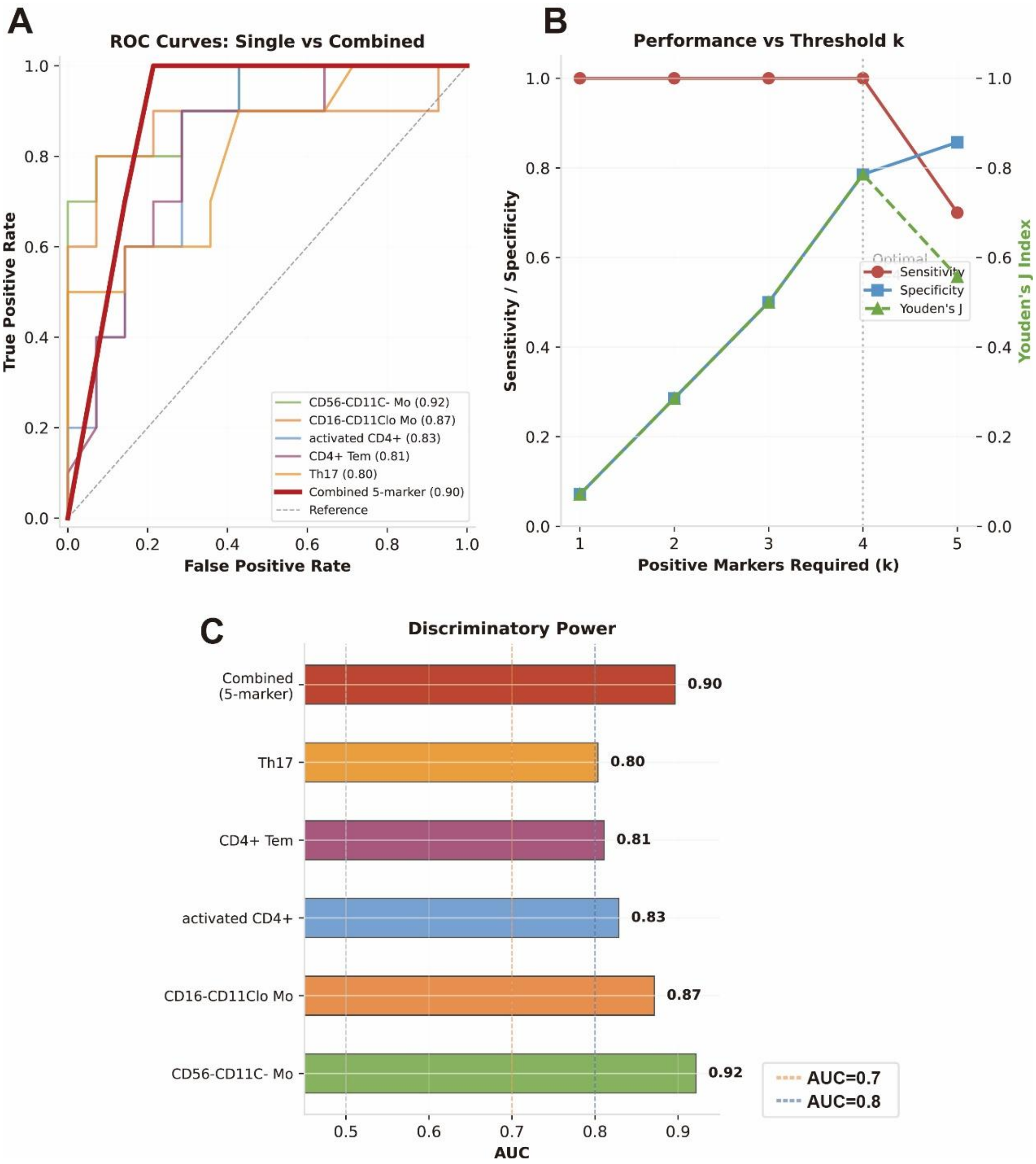

Supplementary Figure S5. A. Composite immune indices across disease groups. B. Innate-adaptive immune rebalancing: Monocyte/T-cell ratio and severity gradient. C. Multi-marker combined discriminatory analysis. (A) ROC curves comparing individual top markers versus combined 5-marker voting score (AUC=0.90) for severe MPP (SMPP-N, n=10) versus mild MPP (MNMPP-N, n=14) discrimination. Individual

marker AUCs: CD56-CD11C- Mo (0.92), CD16-CD11Clo Mo (0.87), activated CD4+ (0.83), CD4+ Tem (0.81), Th17 (0.80). (B) Sensitivity, specificity, and Youden's J index as a function of required positive marker threshold (k); optimal cutoff at >=4 positive markers yields 100% sensitivity and 78.6% specificity. (C) Comparative discriminatory power. Optimal individual cutoff points (Youden method): CD56-CD11C- Mo >14.80% (Sens 80.0%, Spec 92.9%); CD16-CD11Clo Mo >15.27% (Sens 80.0%, Spec 92.9%); activated CD4+ >3.19% (Sens 90.0%, Spec 71.4%); CD4+ Tem >7.74% (Sens 90.0%, Spec 71.4%); Th17 >0.64% (Sens 50.0%, Spec 100%).

(6) Supplementary Material S6

Supplementary Figure S6. Grouping Diagram

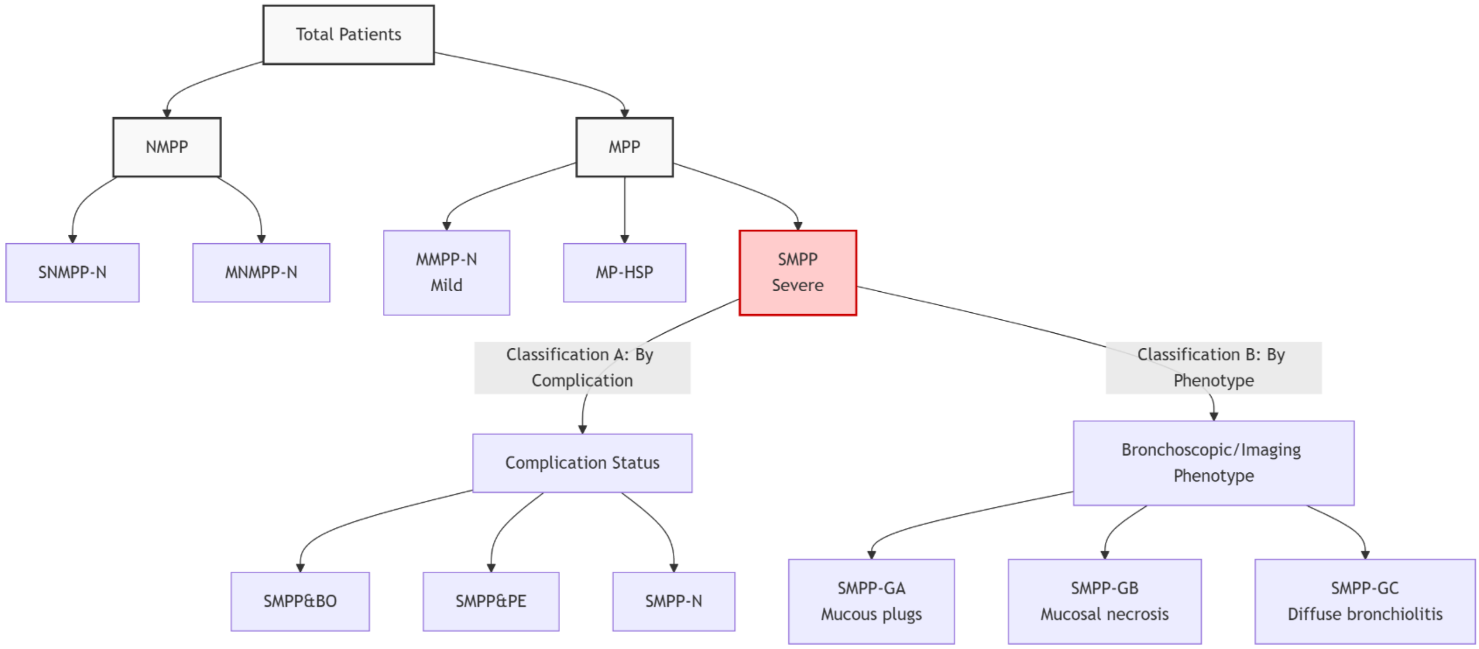

(7) Supplementary Material S7

Group 1	Group 2	Group 3
MPP	MMPP-N	
	SMPP	SMPP-N<br>SMPP&BO<br>SMPP&PE<br>SMPP-GA<br>SMPP-GB<br>SMPP-GC
	MP-HSP	
NMPP	SNMPP-N<br>MNMPP-N	

Supplement Table S1. Note: *Mycoplasma pneumoniae* pneumonia (MPP); Mild *Mycoplasma pneumoniae* pneumonia without complication (MMPP-N); Severe *Mycoplasma pneumoniae* pneumonia (SMPP); *Mycoplasma pneumoniae* pneumonia with Henoch-Schonlein purpura (MP-HSP); Severe *Mycoplasma pneumoniae* pneumonia without complication (SMPP-N); Severe *Mycoplasma pneumoniae* pneumonia with bronchiolitis obliterans (SMPP&BO); Severe *Mycoplasma pneumoniae* pneumonia with pleural effusion (SMPP&PE); SMPP-group A (SMPP-GA): predominant imaging findings of large consolidation, with airway mucus hypersecretion leading to mucus plugging or plastic bronchitis under bronchoscopy, no obvious mucosal necrosis, and no significant elevation in rou-tine laboratory markers; SMPP-group B (SMPP-GB): some children presented with markedly increased C-reactive protein (CRP), and bronchoscopy primarily revealed mucosal erosion and necrosis, often accompanied by mucus plugs; SMPP-group C (SMPP-GC): imaging mainly showed diffuse bronchiolitis, often with hypoxemia, and bronchoscopy typically revealed scant airway mucus secretion without mucosal ero-sion or necrosis; Non-*Mycoplasma pneumoniae* pneumonia (NMPP); Severe Non-*Mycoplasma pneumoniae* pneumonia without complication (SNMPP-N); Mild Non-*Mycoplasma pneumoniae* pneumonia without complication (SNMPP-N).

(8) Supplementary Material S8

SPECIFICITY	FLUOROCHROME	PURPOSE	AB CLONE
Live/Dead	n/a	viability	n/a
CD3	AF488	T cell	UCHT1
CD4	BV510	CD4	SK3
CD8	BV650	CD8	RPA-T8
CD11c	PE-Cy5	mDC	Bly6
CD14	BUV395	monocyte	MΦP9
CD16	BUV496	monocyte	3G8
CD20	BV450	B cells	L27
CD25	BV711	activated CD4	2A3
CD38	BV421	activation	HIT2
CD45RA	PerCP-Cy5.5	naïve	HI100
CD56	BUV737	NK	NCAM 16.2
CD123	AF700	pDC	32703
CD127	APC-eF780	Treg	RDR5
CD183/CXCR3	PE	Th subset	1C6
CD185/CXCR5	PE-Cy7	Th subset	RF8B2
CD194/CCR4	BV605	Th subset	L291H4
CD194/CCR6	BV785	Th subset	G034
CD197/CCR7	PE-CF594	central/effector	150503
CCR10	APC	Th subset	314305
HLADR	BUV661	DC	G46-4

Supplement Table S2. Note: DC: dendritic cell, mDC: myeloid dendritic cell, pDC: plasmacytoid dendritic cell, Treg: T regulatory cell, Th: T helper cell, NK: natural killer cell.
